# Alternative Splicing and Transcription Elongation in Plants

**DOI:** 10.3389/fpls.2019.00309

**Published:** 2019-03-26

**Authors:** Micaela A. Godoy Herz, Alberto R. Kornblihtt

**Affiliations:** ^1^ Departamento de Fisiología, Biología Molecular y Celular, Facultad de Ciencias Exactas y Naturales, Universidad de Buenos Aires (UBA), Buenos Aires, Argentina; ^2^ Instituto de Fisiología, Biología Molecular y Neurociencias (IFIBYNE), CONICET-UBA, Buenos Aires, Argentina

**Keywords:** alternative splicing, transcription, plants, RNAPII, light

## Abstract

Alternative splicing and transcription elongation by RNA polymerase II (RNAPII) are two processes which are tightly connected. Splicing is a co-transcriptional process, and different experimental approaches show that splicing is coupled to transcription in *Drosophila*, yeast and mammals. However, little is known about coupling of transcription and alternative splicing in plants. The kinetic coupling explains how changes in RNAPII elongation rate influence alternative splicing choices. Recent work in *Arabidopsis* shows that expression of a dominant negative transcription elongation factor, TFIIS, enhances exon inclusion. Furthermore, the *Arabidopsis* transcription elongation complex has been recently described, providing new information about elongation factors that interact with elongating RNAPII. Light regulates alternative splicing in plants through a chloroplast retrograde signaling. We have recently shown that light promotes RNAPII elongation in the affected genes, while in darkness elongation is lower. These changes in transcription are consistent with elongation causing the observed changes in alternative splicing. Altogether, these findings provide evidence that coupling between transcription and alternative splicing is an important layer of gene expression regulation in plants.

## Transcription and Alternative Splicing

Transcription in eukaryotes is functionally coupled to mRNA maturation (Saldi et al., 2017), which includes capping, splicing, and polyadenylation. Alternative splicing and transcription elongation by RNA polymerase II (RNAPII) are two processes which are tightly connected. Splicing is a co-transcriptional process, and different experimental approaches show that splicing is coupled to transcription in *Drosophila*, yeast, and mammals. The first evidence that splicing occurs co-transcriptionally was observed by early electron microscopy in nascent transcripts containing splicing loops in *Drosophila* embryos (Beyer and Osheim, 1988). Tilgner and collaborators have shown in a genome-wide deep-sequencing analysis of nascent transcripts that, in human genes, splicing associated to chromatin is mostly co-transcriptional. To investigate this, the authors developed a method that allows to assess the degree of splicing completion around internal exons. They found that in polyadenylated RNAs from cytosolic fractions splicing is almost fully completed. On the other hand, in nuclear RNAs, the majority of human introns are excised while they are still associated with chromatin (Tilgner et al., 2012). This supports the idea that splicing occurs during transcription.

More recently, a work performed in yeast demonstrates that the process of splicing coincides with intron exit from RNAPII: with high-resolution sequencing techniques, nascent RNAs from 87 yeast genes were analyzed (about one third of intron containing genes in this organism). An adaptor was ligated to each 3′ end: this enables to establish the exact position of RNAPII in every nascent RNA molecule. The authors found that, when RNAPII has transcribed about 26–27 nucleotides downstream of the 3′ splice site, the splicing catalysis begins: this work is a high-resolution evidence that splicing *in vivo* is co-transcriptional (Carrillo Oesterreich et al., 2016).

Furthermore, multiple evidences for coupling between transcription and alternative splicing have been obtained working with mammalian culture cells (Cramer et al., 1997, 1999; Kadener et al., 2001; Nogués et al., 2002; de la Mata et al., 2003). It is important to point out that we refer to a functional coupling, that is, properties of the splicing reactions itself are affected by the transcriptional process, not only because they occur at the same time and space: coupling occurs if the splicing reactions depend on transcription and transcription depends on splicing (Lazarev and Manley, 2007). However, little is known about coupling of transcription and alternative splicing in plants.

Two mechanisms, which are not mutually exclusive, have been proposed to explain the nature of coupling between transcription and alternative splicing: recruitment coupling and kinetic coupling. The first mechanism involves recruitment of splicing factors by the transcription machinery. The kinetic coupling explains how changes in RNAPII elongation rate influence alternative splicing choices (de la Mata et al., 2003; Dujardin et al., 2014; Fong et al., 2014).

The recruitment coupling focuses on recruitment of splicing factors by the transcription machinery: RNAPII largest subunit contains a carboxy-terminal domain (CTD). In animals and plants, the CTD is composed of a number of repeats of a consensus heptad YSPTSPST that can be subject to different post-translational modifications in their residues. The number of repeats varies among species: the human CTD has 52 repeats, while in plants the CTD has 34 repeats (Koiwa et al., 2004). Modifications of the CTD regulate the affinity for factors involved in capping, 3′ end processing and alternative splicing (McCracken et al., 1997). One of them is phosphorylation, and the results that phosphorylation elicits depend on the involved amino acids. For example, phosphorylation of serine 5 is associated with recruitment of enzymes involved in 5′ capping, while phosphorylation of serine 2 participates in 3′ end processing (Kim et al., 2004). The CTD phosphorylation patterns determinate the recruitment of splicing factors to transcription sites (Moore and Proudfoot, 2009; Perales and Bentley, 2009).

The kinetic coupling explains how changes in RNAPII elongation rate influence alternative splicing. The first direct evidence of kinetic coupling *in vivo* was achieved working with a RNAPII mutant in human culture cells, which encodes a point mutation that produces a slow transcription rate. Transcription by the slow RNAPII mutant favors exon inclusion in a reporter minigene compared to wild-type RNAPII (de la Mata et al., 2003). In this reporter minigene (that encodes fibronectin exon EDI), there are two 3′ splice sites: the upstream 3′ splice site is weak, while the downstream one is strong (this means that it is more adjusted to the consensus sequence compared to the weak one). If RNAPII elongation rate is fast, both sites are presented to the splicing machinery at the same time, and the strong one is recognized by the splicing machinery more efficiently: this produces exon skipping. On the other hand, if RNAPII transcription rate is slow, the splicing machinery will recognize the first, weak 3′ splice site, and afterwards, the strong 3′ splice site. This, in turn, leads to exon inclusion. This, however, does not mean that the first intron will necessarily be eliminated before the second one: once “commitment” to include the exon is achieved, the order of intron removal becomes irrelevant (de la Mata et al., 2010). Interesting information has been obtained from single-molecule imaging analyses of constitutive and alternative splicing to identify the intracellular sites of splicing (Vargas et al., 2011). This work demonstrates that, although catalysis of constitutive splicing is co-transcriptional, catalysis of alternative splicing occurs post-transcriptionally in a group of studied events, which does not rule out that recruitment of splicing factors needed for those alternative events takes place co-transcriptionally.

It is important to point out that in most cases, a slow RNAPII produces exon inclusion. However, in some examples, a slow RNAPII promotes exon skipping by allowing the recruitment of negative factors to the splice sites (Dujardin et al., 2014; Fong et al., 2014). In both cases, transcription elongation regulates alternative splicing.

## Elongation Factors: Transcription Factor II S

Changes in RNAPII elongation rate can be caused by different factors: histone chaperones and nucleosome remodelers can facilitate RNAPII movement through chromatin; some DNA sequences may be more difficult to transcribe than others owing to their DNA topology; and histone marks can tighten or loosen DNA binding around nucleosomes (for a detailed review on alternative splicing and chromatin modifications, see Luco et al., 2011). Furthermore, the balance between pausing and activating transcription elongation factors modulates the level of pausing (Jonkers and Lis, 2015).

Transcription factor II S (TFIIS) is an elongation factor required for RNAPII processivity that stimulates RNAPII to reassume elongation after pausing (Fish and Kane, 2002). Recently, a dominant negative TFIIS *Arabidopsis* plant was constructed by replacing two key amino acids responsible for its stimulatory activity by alanines (Dolata et al., 2015). TFIIS mutant plants show a range of developmental and morphological defects. Changes in alternative splicing were analyzed in TFIIS mutant plants: these plants show some alternative splicing defects. The authors found that, out of 284 events analyzed, 62 were altered. In 45 of them, the observed changes correspond to what would be predicted by the kinetic model (preferential selection of upstream 5′ splice sites and enhanced exon inclusion in the case of exon skipping alternative splicing events) (Dolata et al., 2015).

## Plant Transcription Elongation Complex

The *Arabidopsis* transcription elongation complex has been recently described, providing new information about elongation factors that interact with elongating RNAPII. Antosz et al. performed reciprocal tagging in combination with affinity purification and mass spectrometry and demonstrated which transcription elongation factors copurify with elongating RNAPII (using an antibody against Ser2-phosphorylated carboxy-terminal domain repeats of RNAPII). They have shown that RNAPII interacts with TFIIS, PAF1-C, FACT, SPT4/5, and SPT6, among other transcription elongation factors. These results are similar to what has been previously observed in yeast (Krogan et al., 2002), suggesting that the elongation factors that associate with elongating RNAPII both in *Arabidopsis* and in yeast are conserved. In addition, the elongation factors mentioned above also copurified with splicing factors and with some spliceosomal complexes, like U1, U2, U5, among others (Antosz et al., 2017). This suggests that an interplay between transcription elongation and splicing regulation is to be expected in plants.

## Light Regulation of Alternative Splicing Through Transcription Elongation

Our laboratory has shown that light regulates plant alternative splicing (Petrillo et al., 2014). *Arabidopsis* seedlings were exposed to light and dark conditions and alternative splicing was studied in a group of alternative splicing events. Light initiates a chloroplast retrograde signal that regulates nuclear alternative splicing of a subset of transcripts, which encode proteins involved in RNA processing (Petrillo et al., 2014). This light effect depends on functional chloroplasts, since the use of drugs that block chloroplast photosynthetic transport chain inhibits the effect of light on alternative splicing.

**Figure 1:**
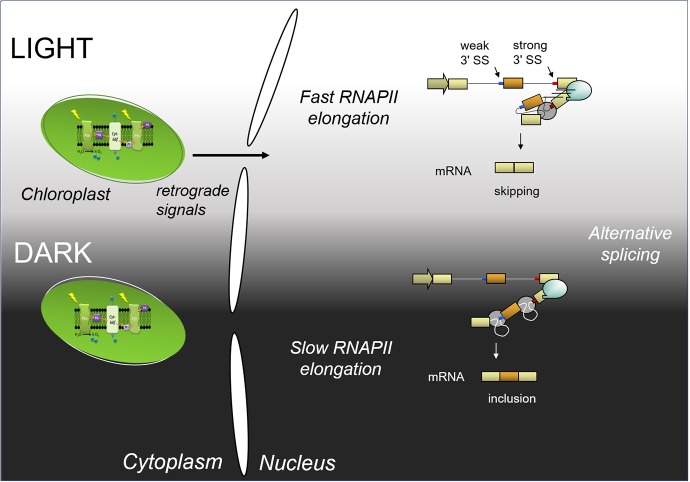
Scheme of light regulation of alternative splicing through the control of transcriptional elongation (adapted from Godoy Herz et al., 2019). Light initiates a chloroplast retrograde signal. In light, RNAPII elongation is fast; both splicing sites (SS) are presented to the splicing machinery at the same time, and the strong one is recognized by the splicing machinery more efficiently: this produces exon skipping. In darkness, RNAPII elongation is slow; the splicing machinery will recognize the first, weak 3′ splice site, and afterwards, the strong 3′ splice site. This, in turn, leads to exon inclusion.

In a recent publication (Godoy Herz et al., 2019), we show that the light control of alternative splicing responds to the kinetic coupling mechanism. Light promotes transcription elongation, while in darkness RNAPII elongation is lower. We show by different experimental approaches that light changes RNAPII elongation, including RNAPII chromatin immunoprecipitation and a modified nascent RNA single molecule intron tracking (SMIT) method, originally described by Carrillo Oesterreich et al. (2016). Briefly, 3′ ends of nascent transcripts were sequenced after treating plants with light and darkness. As a result, the detected 3′ ends correspond to RNAPII positions. Comparison of the densities of 3′ ends along different genes show changes in elongation or processivity in light and dark. This is consistent with elongation causing the observed changes in alternative splicing. Furthermore, the light control on alternative splicing is abolished in the TFIIS mutant plants previously described (Dolata et al., 2015): TFIIS mutant plants do not respond to light signaling on a group of studied alternative splicing events. Thus, light regulates alternative splicing through control of transcriptional elongation. A scheme that summarizes these findings is shown in Figure 1.

The mechanism that explains how light promotes RNAPII elongation remains unknown: as a first attempt to study a possible mechanism mRNA levels of different elongation factors were measured. Although mRNA levels of TFIIS do not change in light and dark, the mRNA levels of the PAF1-C subunits CDC73 and ELF7 increase in light-treated plants. This opens the possibility that an increase in RNAPII elongation is caused by an increase in elongation factor expression (Godoy Herz et al., 2019).

We found that treating *Arabidopsis* seedling with the histone deacetylase inhibitor trichostatin A (TSA) mimics the effect of light on alternative splicing, which we interpreted as a consequence of higher RNAPII elongation due to chromatin relaxation caused by histone hyperacetylation. However, histone acetylation does not participate in the mechanism involved, as has been demonstrated by chromatin immunoprecipitation and Western blot experiments. A role for chromatin modifications on alternative splicing has been intensively studied in mammalian cells (Kadener et al., 2001; Nogués et al., 2002; Alló et al., 2009; Schor et al., 2013; Fiszbein et al., 2016). Chromatin participation on the regulation of alternative splicing in plants remains an interesting field to investigate.

## Perspectives

The results summarized in Figure 1 point at alternative splicing regulation by transcription elongation as a mechanism to respond to an environmental stimulus. Furthermore, they provide evidence that coupling between transcription and alternative splicing is important for a whole organism (plants) to respond to environmental cues (light). It is interesting to point out that plants are a model organism suitable for this kind of experimental approaches and that there is still plenty to learn about gene expression regulation from this kingdom. More efforts will be needed to understand the mechanisms involved in light-mediated RNAPII elongation: How does light affect transcription elongation? How do changes in transcription elongation modulate alternative splicing? Nevertheless, the experimental system described here provides a mean to investigate the mechanism behind the proposed regulation of alternative splicing by transcription elongation in whole organisms.

## Author Contributions

MGH and AK wrote the manuscript.

### Conflict of Interest Statement

The authors declare that the research was conducted in the absence of any commercial or financial relationships that could be construed as a potential conflict of interest.
